# Implementation of an Electronic Endoscopic Stent Register in a District General Hospital in the National Health Service (NHS), United Kingdom

**DOI:** 10.7759/cureus.110430

**Published:** 2026-06-08

**Authors:** Ahmed Abdelwahed, Radhakrishnan Ganesh, Stephen McNally

**Affiliations:** 1 Upper GI and General Surgery, Raigmore Hospital, NHS Highland, Inverness, GBR

**Keywords:** endoscopic retrograde cholangiopancreatography (ercp), plastic biliary stent, quality improvement and patient safety, stent register, stent-related complications

## Abstract

Introduction

Serious and life-threatening complications can occur from the delayed removal of biliary stents. A robust system is needed to ensure the timely removal of stents. We aimed to evaluate our practice of using a stent register to remove stents on time and identify system failures that contribute to missed stent removals.

Method

A new electronic stent register was introduced in our endoscopy service of a busy district general hospital under the National Health Service (NHS) in the United Kingdom. This register was initiated to support the tracking of patients who had biliary stents placed to ensure that this is removed on time. We analysed the usefulness of our register in managing patients who had a biliary stent placed. Data was extracted from the database after a 20-month data collection period. Collected data included stent type and follow-up plans. The electronic record was examined for clinical course and evidence of removal.

Results

Over the study period, 414 patients underwent endoscopic retrograde cholangiopancreatography (ERCP), and 89 patients had stents placed. After clinical review, 82 cases were managed appropriately. In seven cases, stents were overdue for removal. The reasons for delay were: patient preference (n=1); relocation out of area (n=2); and inadequate follow-up (n=4). The register also identified patients with oesophageal and lumen-apposing metal stents, all of whom had appropriate follow-up and removal plans documented.

Conclusion

Inadequate follow-up documentation and unclear responsibility for ongoing surveillance were identified in the majority of genuinely overdue cases. Implementation of an electronic stent register successfully identifies patients with overdue stent removal or exchange. Successful implementation of a stent register requires regular clinical oversight and consistent staff engagement.

## Introduction

The placement of stents is an important therapeutic option in endoscopic retrograde cholangiopancreatography (ERCP) for both benign and malignant disease [1]. Indications for benign biliary stenting include biliary stricture, obstructing common bile duct (CBD) stones, and bile leaks. Stents require carefully planned follow-up and timely exchange or removal to prevent encrustation or blockage, which can lead to obstructive jaundice or cholangitis.

Delayed removal of stents may occur due to patient-related factors, such as not implementing endoscopist instructions [2], or physician-related factors, including lack of proper documentation and follow-up [3]. Rapid resolution of symptoms can also make patients forget the presence of a stent [3].

Failure to remove stents on time can lead to serious adverse events. Although some forgotten biliary stents remain asymptomatic, many can present with clinically significant complications such as cholangitis [4-7], choledocholithiasis, stentolithiasis, duct stricture, and stent migration [3]. These problems occur more frequently in elderly patients [6]. Plastic biliary stents are also susceptible to occlusion, which may develop at any time from a few days to several months after insertion [8]. Stent-related complications require additional interventions and increase the number of hospital admissions [2,6,7,9].

Temporary endoscopic stents require structured and timely surveillance because prolonged dwell time is associated with multiple recognised complications, including cholangitis, polymicrobial infection, stent occlusion, migration, tissue overgrowth, and technically challenging retrieval [2-4]. Current guidance recommends that plastic biliary stents be exchanged in about three to six months to avoid these complications [2,10,11]. Given these considerations, a reliable and accurate system is essential to monitor stents inserted and the due time for exchange or removal. Electronic stent registries have therefore been recommended as a key safety tool to reduce the incidence of forgotten stents and prevent associated morbidity [3,5,12]. In this study, the term “Electronic Stent Register” refers to a structured patient tracking and governance system incorporated within the Endoscopy Management System rather than an electronically traceable stent device. The novelty of this study lies in the evaluation of a structured electronic follow-up process designed to identify patients at risk of delayed stent management within routine clinical practice.

This study was conducted to evaluate the performance of a newly implemented electronic stent register in order to assess its accuracy and identify system gaps in order to improve patient safety for those undergoing biliary stenting. The primary aim of this study was to evaluate the feasibility of the electronic stent register to identify patients with potentially overdue temporary endoscopic stents requiring further clinical review. Secondary aims included assessment of the rate of appropriate follow-up and identification of workflow-related factors contributing to delayed stent management.

## Materials and methods

This study involved a retrospective review of de-identified human patient data and was conducted as a service evaluation/quality improvement project. In accordance with local institutional governance guidance, formal research ethics committee approval was deemed unnecessary for this service evaluation project. Individual patient consent was waived due to the retrospective use of anonymized data.

The study was conducted at Raigmore Hospital, a secondary care District General Hospital in the National Health Service (NHS) Highland, in Inverness, Scotland, United Kingdom, serving a mixed urban and rural referral population within the NHS Highland, which covers a geographically large area of approximately 35,500 square kilometres and a population of 330,000. The institution manages complex biliary disease and hepato-pancreato-biliary malignancy and performs approximately 300 ERCP procedures annually, providing services for both local patients and tertiary hepatopancreatic biliary referrals.

The responsibility of managing the biliary stent was under the clinicians who requested the procedure. Each ERCP report contains instructions regarding the time period during which the stent has to be removed or replaced. This system did not ensure that the biliary stent was managed appropriately, and patients presented at a later stage with stent-related complications. A novel electronic stent register was thus introduced in our endoscopy unit’s electronic management system (EMS) (MEDILOGIK Ltd., Worcester, United Kingdom) to avoid mismanagement of biliary stents.

The electronic stent register was an internally developed electronic tracking database incorporated within the Endoscopy Management System rather than a standalone software package. When a patient was scheduled for an endoscopic procedure, demographic details were entered into EMS as part of the routine booking process. Following ERCP and stent insertion, information recorded within the endoscopy report was automatically incorporated into the electronic stent register as part of the Endoscopy Management System, eliminating the need for data entry into a separate software application. Information captured included patient identifiers, stent type, date of insertion, intended follow-up, and planned intervention (stent removal, exchange, imaging, or clinical review). The information was captured during routine procedure documentation. Stents inserted as a planned permanent intervention did not appear within the register. The register did not generate fully automated alerts but functioned as a governance and tracking tool requiring regular clinical review to identify patients potentially overdue for follow-up. The register was reviewed routinely during the endoscopy governance and clinical follow-up review process. 

Although the primary focus of this study was biliary stent surveillance, the electronic register was designed to capture all temporary endoscopic stents requiring planned follow-up, including oesophageal stents and lumen apposing metal stents (LAMS). These non-biliary stents were presented as secondary observations within the current analysis.

We retrospectively reviewed our practice of managing biliary stents 20 months after the introduction of a biliary stent registry. We assessed the efficacy of this system in making sure that biliary stents were managed appropriately within the time frame after a 20-month implementation period. All data within the stent register were extracted, and a clinical review of case notes was performed. Data were collected on the type of stent placed, the planned follow-up schedule, and the procedure.

Supplemental data were extracted from the electronic patient record to confirm that clinical follow-up and plans for stent removal or exchange were completed. Stents were regarded as overdue if they were more than four weeks beyond their planned exchange or removal date. 

## Results

Over a 20-month period, the electronic stent register listed 93 active patients requiring ongoing intervention, predominantly ERCP cases (n=89), with one oesophageal stent and three LAMS. Most biliary stents were plastic double-pigtail stents, with a smaller number of metal and straight plastic stents (Table 1).

**Table 1:** Types of endoscopic stents recorded into the electronic stent register

Stent type:	Number
Double pigtail plastic stents	75
Single pigtail plastic stents	10
Straight plastic stents	1
Covered metal biliary stents	2
Fully covered metal biliary stent	1
Lumen apposing metal stents	3
Covered oesophageal stent	1

Planned follow-up for stents placed during ERCP and documented at the time of stent insertion included: repeat ERCP stent removal or exchange; abdominal radiography (for pancreatic stents); gastroscopy for removal; or clinical review (Table 2). Follow-up interval varied considerably, particularly for plastic biliary stents, ranging from one to six months. Some metal stents were listed for a 12-month review. 

**Table 2:** Documented follow-up plans for patients entered into the electronic stent register

Follow-up plan	Count
Abdominal X-ray	8
Removal at endoscopic retrograde cholangiopancreatography	63
Gastroscopy for stent removal	10
For clinical review	12

**Figure 1:**
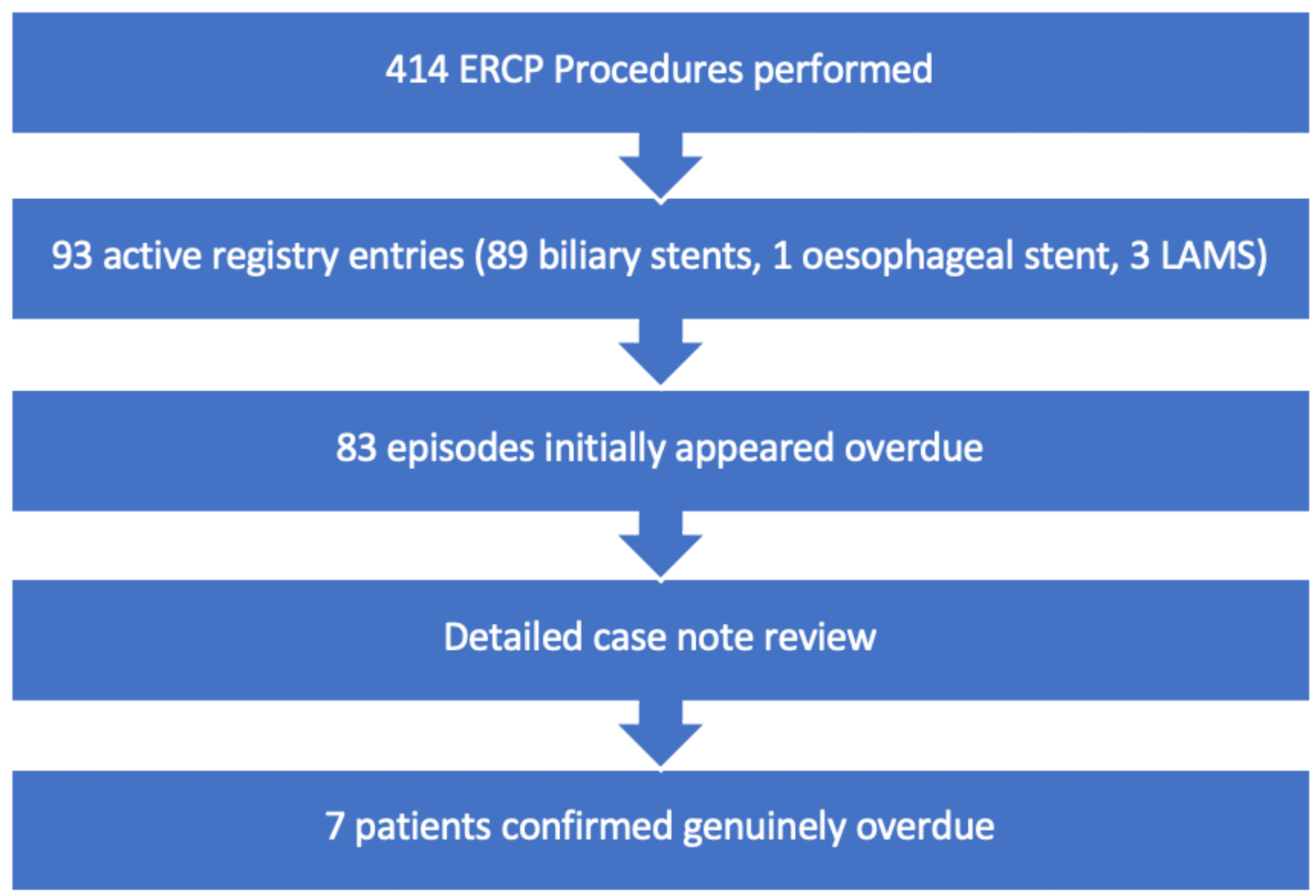
Flow diagram of registry review process and identification of overdue stent cases ERCP: endoscopic retrograde cholangiopancreatography; LAMS: lumen apposing metal stents

During the 20-month study period, 414 ERCP procedures were performed. Temporary biliary stents were inserted in 89 patients. The electronic register also included one oesophageal stent and three LAMS, giving a total of 93 active registry entries requiring follow-up. Following the removal of duplicate entries and the review of electronic records, 83 follow-up episodes initially appeared overdue within the register. Subsequent clinical review identified that many had undergone timely stent removal at surgery or endoscopy, and detailed case-note review confirmed that only seven patients were genuinely overdue for planned stent removal or exchange. Figure 1 summarises the registry review process and the identification of the genuinely overdue cases. 

Reasons in the seven patients who were genuinely overdue included (i) patient declined follow-up (n=1), (ii) Patient relocation (n=1), (iii) refusal to transfer from a remote location (n=1), and (iv) the absence of a formal discharge letter with a follow-up plan (n=4). In cases without a discharge letter, either no follow-up was arranged, the intended timeframe for stent removal was not documented within the immediate discharge letter, or responsibility for follow-up was unclear. Table 3 summarises the genuinely overdue cases identified during the registry review. 

**Table 3:** Summary of genuinely overdue cases ERCP: endoscopic retrograde cholangiopancreatography

Case	Planned follow-up	Cause of delay	Corrective action taken
1	Repeat ERCP for stent removal	Patient declined follow-up procedure	Not Applicable
2	Repeat ERCP for stent removal	Patient relocated out of the area	Not Applicable
3	Repeat ERCP for stent removal	Sent refused transfers from a remote location	Organised a gastroscopy and stent removal in remote location
4	Repeat ERCP for stent exchange	No formal discharge letter with a follow-up plan.	Listed for a repeat ERCP
5	Repeat ERCP for stent removal	No formal discharge letter with a follow-up plan.	Listed for a repeat ERCP
6	Repeat ERCP for stent removal	No formal discharge letter with a follow-up plan.	Listed for a repeat ERCP
7	Repeat ERCP for stent exchange	No formal discharge letter with a follow-up plan.	Listed for a repeat ERCP

The single oesophageal stent had been removed within the expected timeframe, and all three LAMS were appropriately managed. 

## Discussion

This study demonstrates that biliary stent follow-up is adequately managed in over 90% of cases, and that an electronic endoscopic stent register can be used to identify those patients with stents that lack adequate follow-up. However, although the register initially identified a large number of apparently overdue episodes, detailed review confirmed that only a small proportion represented genuinely overdue cases. This reflects the fact that the register functions primarily as a screening tool and requires manual clinical review of electronic records and case notes to confirm whether follow-up had already taken place elsewhere or alternative management plans had been made. While this demonstrates the usefulness of the register in identifying patients requiring further review, it also highlights that the system is not sufficient as a stand-alone safety mechanism without regular clinician-led oversight.

Several strategies have been considered to facilitate the timely removal of stents. Clear communication with patients regarding the planned date of stent removal is advised. Routine review of all ERCPs at three months was done in some centres to identify people who are due for stent removal [6,13]. Use of electronic reminders can prevent forgotten urethral stents [3,14]. Biodegradable stents, which do not need further removal, reduce the need for reintervention, although they will not be applicable for all clinical scenarios [15]. 

The European Society of Gastrointestinal Endoscopy recommends maintaining a biliary stent registry to minimise stent-related complications [5,12]. This should function as a prospective registry of all biliary stents and facilitate the recall of patients for stent removal or exchange. A register can reduce stent-related sepsis by decreasing the incidence of stent occlusion through timely stent replacement [16].

Our findings support the utility of an electronic stent register. Although the register initially appeared to show a high number of overdue cases, detailed review confirmed that only seven patients were genuinely overdue. This highlights the importance of careful data review to prevent a true lapse in follow-up for patients with temporary stents. The register also successfully captured all LAMS cases, which carry significant risks such as buried stent syndrome if they are left in situ beyond the recommended time. This demonstrates its value as an effective safety mechanism to prevent such complications, particularly in endoscopy units where multiple teams share responsibility for follow-up. However, our results demonstrate the need for active clinician review of cases on the register to ensure action is taken when appropriate, and the existence of a register in itself does not minimise patient risk without clinical review of register cases. 

One of the most significant findings of this study was the absence of a formal discharge letter in the majority of cases confirmed to be genuinely overdue. In these patients, the intended timeframe for removal was not documented on discharge paperwork, no follow-up appointment was generated, and responsibility for arranging follow-up was not clearly assigned. Previous studies have similarly highlighted that inadequate communication and poor documentation are key contributors to retained biliary stents and associated complications [3,17]. Our findings reinforce that discharge letters function as a critical safety checkpoint, ensuring that the follow-up plan is explicit and traceable. Without clear documentation, the risk of miscommunication increases substantially. 

A wider review of unit practice over this time period identified paper records as a further potential gap in data recording. ERCP cases performed outside the Endoscopy unit (in theatre, under general anaesthesia) were typically recorded as an operation note and were not always transferred onto the Endoscopy Management System and hence not transferred into the electronic register, creating tracking gaps. Strengthening the link between theatre and endoscopy records is essential to ensure that all stents are captured and appropriately monitored.

Successful implementation of a stent register requires ongoing staff engagement, clear allocation of responsibility, and protected clinical time for regular review of cases. Although data entry into the register was incorporated into routine procedure documentation, accurate follow-up still depended on consistent updating of records and active review by clinical teams. In our experience, the register functioned most effectively when responsibility for follow-up and review was clearly defined within the endoscopy service governance structure.

The medicolegal responsibility for forgotten stents and their complications has been shown to fall predominantly on the endoscopist [14]. While an electronics stent registry is a valuable safety tool, it cannot prevent adverse outcomes in isolation. Routine, protected clinical time is required for register review and to pull data from a number of sources. 

Geographical factors also contributed to delays in a small number of cases. Limited access to specialist endoscopy services, particularly for patients living in remote or rural areas, has been recognised as a barrier to timely follow-up. Early communication and coordinated planning with local healthcare providers may help mitigate these challenges and ensure that patients can access appropriate stent surveillance and removal within recommended timeframes. 

This study has a number of limitations. It was a retrospective study, and therefore the findings were dependent on the accuracy and completeness of the existing electronic records. In addition, as this was a single-centre study carried out in a district general hospital, the findings may not fully reflect practice in other units with different referral pathways or electronic systems. The study also covered the early phase of introducing the electronic stent register, and variation in staff familiarity with the system may have affected the consistency of data entry. Finally, some ERCP procedures performed in theatre under general anaesthesia were documented separately from the endoscopy reporting system and were not always transferred onto the electronic register, meaning that a small number of cases may not have been included in the database. 

Overall, this study supports the role of an electronic stent registry as an important safety tool in endoscopy practice. In line with existing literature and guideline recommendations, a structured tracking system can substantially reduce the risk of forgotten stents and their associated complications. However, our findings highlight that the effectiveness of such a system relies on three essential components: accurate and complete documentation at discharge, timely uploading of data into the registry, and routine clinical-led review of entries. Addressing these areas through mandatory discharge process, staff training, and protected time for regular review would significantly strengthen patient safety and ensure more reliable management of temporary endoscopic stents. 

## Conclusions

An electronic stent register serves as a tool for monitoring temporary endoscopic stents and identifying patients at risk of missed follow-up. Its effectiveness, however, depends on accurate data entry, consistent updating, and regular clinical review. In this study, the register identified patients with delayed follow-up and highlighted weaknesses in documentation and follow-up planning. Further studies would be required to determine whether implementation of such systems reduces stent-related complications, hospital admissions, or other clinical outcomes.
